# Automated Computer Vision-Enabled Manufacturing of Nanowire Devices

**DOI:** 10.1021/acsnano.2c08187

**Published:** 2022-09-26

**Authors:** Teja Potočnik, Peter J. Christopher, Ralf Mouthaan, Tom Albrow-Owen, Oliver J. Burton, Chennupati Jagadish, Hark Hoe Tan, Timothy D. Wilkinson, Stephan Hofmann, Hannah J. Joyce, Jack A. Alexander-Webber

**Affiliations:** † Department of Engineering, University of Cambridge, 9 JJ Thompson Avenue, Cambridge CB3 0FA, United Kingdom; ‡ Australian Research Council Centre of Excellence for Transformative Meta-Optical Systems, Department of Electronic Materials Engineering, Research School of Physics and Engineering, The Australian National University, Canberra ACT 2600, Australia

**Keywords:** nanowires, computer vision, automation, nanofabrication, microscopy

## Abstract

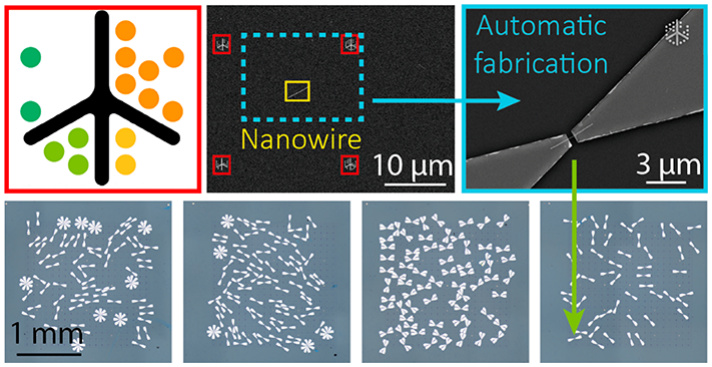

We present a high-throughput method for identifying and characterizing individual nanowires and for automatically designing electrode patterns with high alignment accuracy. Central to our method is an optimized machine-readable, lithographically processable, and multi-scale fiducial marker system—dubbed LithoTag—which provides nanostructure position determination at the nanometer scale. A grid of uniquely defined LithoTag markers patterned across a substrate enables image alignment and mapping in 100% of a set of >9000 scanning electron microscopy (SEM) images (>7 gigapixels). Combining this automated SEM imaging with a computer vision algorithm yields location and property data for individual nanowires. Starting with a random arrangement of individual InAs nanowires with diameters of 30 ± 5 nm on a single chip, we automatically design and fabricate >200 single-nanowire devices. For >75% of devices, the positioning accuracy of the fabricated electrodes is within 2 pixels of the original microscopy image resolution. The presented LithoTag method enables automation of nanodevice processing and is agnostic to microscopy modality and nanostructure type. Such high-throughput experimental methodology coupled with data-extensive science can help overcome the characterization bottleneck and improve the yield of nanodevice fabrication, driving the development and applications of nanostructured materials.

By virtue of their geometries and material properties, devices based on isolated nanomaterial structures, including single crystal domains, grain boundaries,^1,2^ heterojunctions,^3^ stacked bilayers,^4^ optically active defects,^5^ or individual nanowires,^6^ have (opto)electronic characteristics which enable applications not possible with conventional bulk materials. When creating a device based on an individual nanostructure, that structure’s exact position needs to be known. Yet, nanostructures, particularly those grown by bottom-up processes in their early stages of development and those dispersed from solution, are typically irregularly distributed and heterogeneous. Fabricating and measuring nanoscale devices based on such randomly dispersed nanomaterials is labor-intensive, involving searching and alignment before manual routing of the electrode layout or manually performing pick-and-place to transfer these nanostructures onto existing electrode configurations.^7^ Among nanomaterials, semiconducting nanowires attract much interest because of their high aspect ratios, conductivity along the length of the nanowire, and potential for quantum confinement across the nanowire diameter.^8^ They are compatible with existing silicon electronics,^9^ can host heterostructures^10^ and bandgap-graded compositions,^6^ and allow for laterally^11^ or vertically integrated device architectures.^12,13^ They are also desirable due to their high surface-to-volume ratio, high mobilities, and tunable direct bandgaps.^14^ However, many of these proof-of-concept devices are difficult to scale up because detailed microscopy and sophisticated processing^15^ are needed to tackle inhomogeneous nanowire distributions and small feature sizes. Improving the throughput of these microscopy and processing techniques is crucial for nanowire device scalability.

Microscopy techniques, such as optical microscopy (OM), atomic force microscopy (AFM), scanning electron microscopy (SEM), and transmission electron microscopy (TEM), are also limited by the instrument tuning and the skill of the operator. Scalable, automated methods are required to improve the efficiency of microscopy techniques, speed up device fabrication processes, and increase throughput. There has been progress with identification of nanowires using automated OM^16,17^ and AFM,^18^ spatially resolved pick-and-place techniques,^19,20^ and semi-automated contacting of multiple nanowires using pre-patterned electrodes.^21^ The general drive toward automation in microscopy^22^ across a range of fields and imaging modalities draws on advances in image analysis,^23−25^ including material structure recognition^26−29^ and automated microscope hardware control.^30^ Here we address the challenge of nanomaterial device prototyping and characterization using high-throughput automated microscopy for precise position determination of automatically identified nanomaterial structures.

One pathway to automate these processes is through the use of fiducial markers, which are artificial patterns, symbols, or images placed in an environment. When a fiducial marker is recognized by the system, it can be used to determine its location, its orientation, or encoded information. They are widely used in augmented reality applications and are used for a variety of computer vision tasks, including object tracking, image calibration, and positioning. In microscopy, they are most commonly used for mechanical drift correction^31^ and microscope stage movement tracking.^32^ This has allowed multi-technique correlation and location of features with micron-scale accuracy using a machine vision camera.^33^ Nanofabrication processes such as lithography and microscopy use markers such as arrays of squares or crosses to aid locating the features on wafers. Fiducial markers in lithography are used to align successive layers of lithographically defined nanostructures, such as electrodes^15^ or antennae.^34,35^ A specific feature can then be reproducibly found with respect to the marker arrays and used to guide subsequent characterization or fabrication steps on the same feature. However, challenges arise when the markers’ dimensions are not compatible with the resolution requirements of the characterization technique and feature size of the nanostructure, or when they do not encode sufficient position information. For example, regular arrays of squares, crosses, or circles are all the same and do not contain any coordinate information, making it difficult to locate nano-features on large wafers.

In this paper we demonstrate a fiducial marker system, LithoTag, which is optimized for lithographic processing and allows position mapping of features at the nanoscale from images at any arbitrary position on a wafer. We apply it to locate and design nanowire devices entirely automatically, with a typical alignment accuracy within 2 pixels, defined by the resolution of the original microscopy image used for automated imaging. The process enables high-throughput nanofabrication and statistically significant material characterization.

The performance of fiducial marker systems can be evaluated using various metrics, including (1) false-positive rate, where a marker is detected where none is present, (2) inter-marker confusion rate, where one marker is mistaken for another, (3) false-negative rate, where a marker is present but is not detected, (4) minimal marker size, which is the pixel size required for accurate detection, and (5) image contrast.^36^ Some fiducial marker patterns are specifically designed to increase accuracy,^37^ while others are developed to minimize false-positive rate.^36,38^ Requirements for a fiducial marker system can also include robustness, high information density, high recognition reliability, and immunity to imaging conditions.

Additionally, there are requirements specific to the nanofabrication processes, such as lithography, development, and lift-off metallization, that need to be considered for nanofabrication applications. The alignment markers themselves need to be unique and similar in size to the nanomaterial features, and they need to retain high resolution throughout the processing steps to achieve high recognition reliability. They should also include features that are easily distinguishable to use as reference points to minimize uncertainties in the alignment. From a computer vision viewpoint, their shape should also be easily distinguishable from the surroundings, provide good material contrast, and have sufficient information density.

Two challenges associated with the patterning of fiducial markers in nanofabrication are the following: (i) Due to the proximity effect, the exposed area becomes larger than expected, and the features of the pattern can become lost, altering the pattern design and causing uncertainty in alignment marker position.^39,40^ (ii) Reading of the marker becomes challenging due to resolution and contrast limitations that depend on feature sizes, materials, and microscopy techniques. Another resolution-limiting process is lift-off, which is used to transfer the pattern by evaporating metal onto a patterned resist and then dissolving the resist to leave only the deposited metal that was in contact with the substrate. For clean lift-off, an undercut profile should be created with the choice of an appropriate resist to avoid deposition on the sidewalls,^41,42^ and the pattern should avoid enclosed spaces (see Supporting Information, including Figures S1–S3). This is a common problem when ridges on the sidewalls are created during the electron beam lithography (EBL) process due to stochastic fluctuations and noise effects.

Therefore, to minimize overexposure and to ensure the pattern retains its shape during lift-off, the main geometrical requirements of the pattern design are the avoidance of enclosed empty spaces within the pattern and that all features are larger than the minimum achievable resolution of both the lithography and microscopy systems in use. For example, square shapes are not appropriate due to resolution limits, which makes them difficult to write and detect at the nanoscale. All of these phenomena become more pronounced with reduced dimensions of the features.^43^

We trialed a number of existing fiducial marker designs for compatibility with nanofabrication processes, as discussed in the Supporting Information. For each of these designs, the lithographically patterned versions failed to maintain fidelity with the original design, due to combinations of the effects described above. These difficulties demonstrated the need for a suitable lithographically processable marker design.

## Results and Discussion

**Figure 1:**
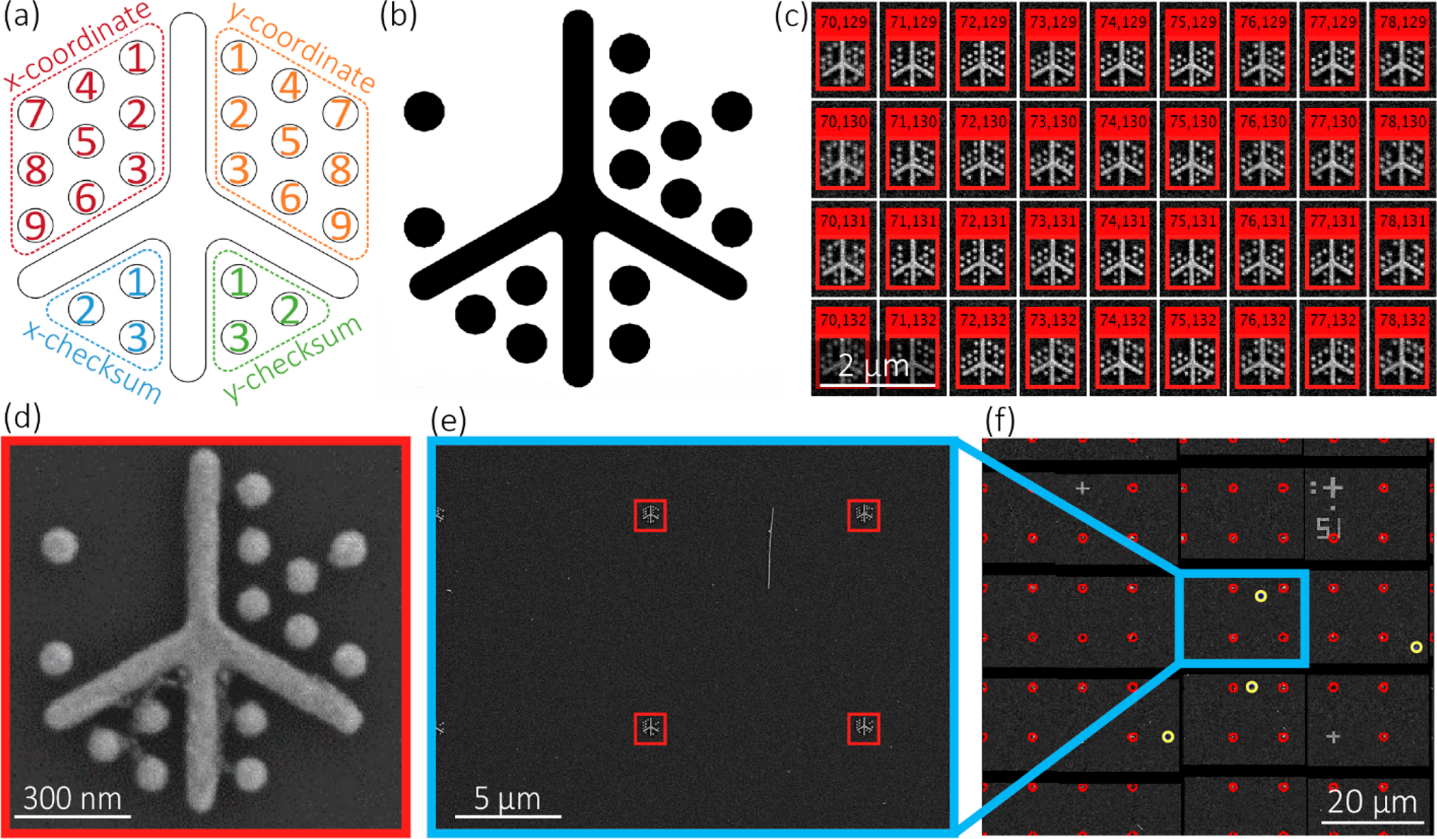
(a) LithoTag fiducial marker design with central spine and radial dots encoding *x*- and *y*-positional coordinates, and encoding checksums for error detection. (b) Example LithoTag fiducial marker pattern. (c) Demonstration of LithoTag detection over a large area. (d) Example of LithoTag fiducial marker as-fabricated with evaporated Ti/Au on a Si/SiO_2_ substrate. (e) Demonstration of LithoTag detection within an arbitrary image from a large map. (f) Demonstration of map image stitching, with red annotations corresponding to LithoTags and yellow to automatically identified nanowires.

Our fiducial marker system, LithoTag (Figure 1a), is optimized for nanofabrication processing, including lift-off, and has high information density to allow precise location mapping from arbitrary microscope images of a patterned substrate. The basic design of the LithoTag marker consists of a cross-type spine, surrounded by circular features arranged in a hexagonal pattern around the spine which correspond to a binary representation of the *x*-coordinates (upper-left) and *y*-coordinates (upper-right). The hexagonal packing ensures a minimum amount of empty space and the highest information density. The spine consists of four arms with aspect ratios and circularities designed to aid recognition. The longest arm of the spine faces upward, which provides information about the orientation of the tag. The design also includes checksums associated with each coordinate for error checking (lower patterns). Checksums are a common method for checking for information errors in a system. In the case of LithoTag, checksums are used for both *x*- and *y*-coordinates to check whether the tag has been read correctly, with no errors. The checksum can be calculated using a cyclic redundancy check from the coordinates.^44,45^ By incorporating one within the tag, it is possible to check for writing or reading errors. This can give information about compatibility with nanofabrication and microscopy processes.

In the context of common lithographic processing techniques, the design of LithoTag describes a locally patterned contrast material (CM, e.g., a physical vapor-deposited metal) upon a uniform global background such as a wafer substrate, where CM is selected to maximize contrast in a given imaging technique (Figure 1b), including OM, SEM or AFM. We fabricated a 205×205 grid of 860-nm-width LithoTags with 10 μm separation and performed automated SEM imaging of the entire grid. InAs nanowires with an average diameter of 30 ± 5 nm were grown in vertical forests on a bulk InAs wafer substrate and subsequently sonicated in isopropanol (IPA) and dispersed onto the LithoTag-marked substrate (see Methods). This leaves a randomly distributed array of individual nanowires. We first focus on LithoTag detection, where each image was analyzed to read LithoTag coordinates. Our computer vision algorithm uses a convolution method to detect LithoTags, which allows for tag recognition even in low-resolution images. The detection uses a template of the LithoTag (Supporting Information Figure S4), which includes the spine and predicted circle locations and is rotated and scaled to the same approximate orientation and scale of the LithoTag in the image. The cross correlation between the two images is then calculated, where the selected peaks in the result allow the tag’s location within the image to be calculated. The design of the LithoTag was chosen for scale invariance. This approach could be made even more robust to variations in substrate orientation and image magnification by implementing a search algorithm to find the configuration of rotation and scale that maximizes the convolutional peaks. Once the position of the tag is known, the expected location of the circles can be inferred. A weighted average of pixel values in the vicinity of each expected circle location is then calculated to read the position and checksum information encoded in the tag. The final step is to perform a checksum calculation to validate the information decoded from the tag. A selection of LithoTags from within an example substrate area shows the LithoTags recognized and correctly identified by the algorithm, shown in Figure 1c. The LithoTags in 100 randomly selected images were hand-labeled for comparison with the computer-labeled LithoTags. Of the 456 LithoTags in these images, only 2 were not identified by our algorithm. Out of the 454 recognized LithoTags, the coordinates of 6 were incorrectly parsed, giving 98.7% detection reliability. The incorrectly parsed LithoTags fail the checksum check. Tag detection also works reliably on blurred images, which is demonstrated in Supporting Information Figure S5. This allows for accurate detection even if the SEM images are out of focus due to non-uniform thickness of the substrate. Figure 1d shows an example LithoTag with a total width of 860 nm using evaporated Ti/Au on an oxidized Si wafer, patterned by EBL. The fabricated LithoTag exhibits minimal deviation from the design. The closer the features are, the greater the potential for proximity effects, and these effects are exacerbated for large contiguous features. This is another advantage of implementing the LithoTags’ dot-based design instead of squares. There is slight bridging between the features seen in some cases, likely due to resist residues after lift-off, but it has been shown that this does not reduce the detection efficiency.

The entire patterned grid was automatically scanned in 9466 SEM images, where the field-of-view for each SEM image captured typically between 4 and 6 LithoTags, to allow accurate position detection and alignment from any image (e.g., Figure 1e). Despite the fact that only 3 LithoTags are required for accurate alignment, additional tags are often present in the image due to the marker grid layout and the aspect ratio of the image. Image stitching of the SEM map is shown in Figure 1f, where all recognized LithoTags are highlighted along with detected nanowires, as described below. The blank areas between the images correspond to regions that were not scanned due to inaccuracies in the stage position of the SEM. The accuracy of the stitching, even with missing areas, indicates the robustness of the technique and that it can mitigate inaccuracies in the mechanical motion of the stage. LithoTag detection could also be incorporated into microscopes with motorized stages to provide real-time positional feedback and distortion correction. These results demonstrate that almost all LithoTags that were imaged were successfully recognized and that the computer vision algorithm correctly identified their coordinates.

To demonstrate the suitability of the LithoTag system, we use it to create hundreds of single InAs nanowire field-effect transistors (FETs). InAs nanowires are also a useful test case because the quasi-one-dimensional nanowire structure presents additional challenges for device fabrication, particularly for lateral side contacts, as required for Hall bar devices and local gate electrodes. InAs nanowires exhibit high room-temperature mobility^46^ and high electron injection velocity, which determine the “on” current in nanoscale FETs, making them suitable for transistor applications, including tunnel field-effect transistors (TFETs).^47^ InAs nanowires exhibit strong surface effects due to charge accumulation at the surface,^48^ resulting in easy Ohmic contact formation^49^ and causing an unconventional decrease in conductivity upon white-light illumination, i.e., negative photoconductivity.^50−55^ They also have a narrow bandgap,^56,57^ which allows for photoresponse tunability and application to a wide range of optoelectronic devices.^6,55^ Their large spin–orbit coupling makes them suitable systems for topological superconductivity.^58,59^ In particular, InAs nanowires have been used from ultraviolet to infrared as photodetectors,^56,60^ in heterojunction photovoltaics,^61,62^ and recently as optoelectronic neuromorphic devices responding to synaptic memory processes.^63^

**Figure 2:**
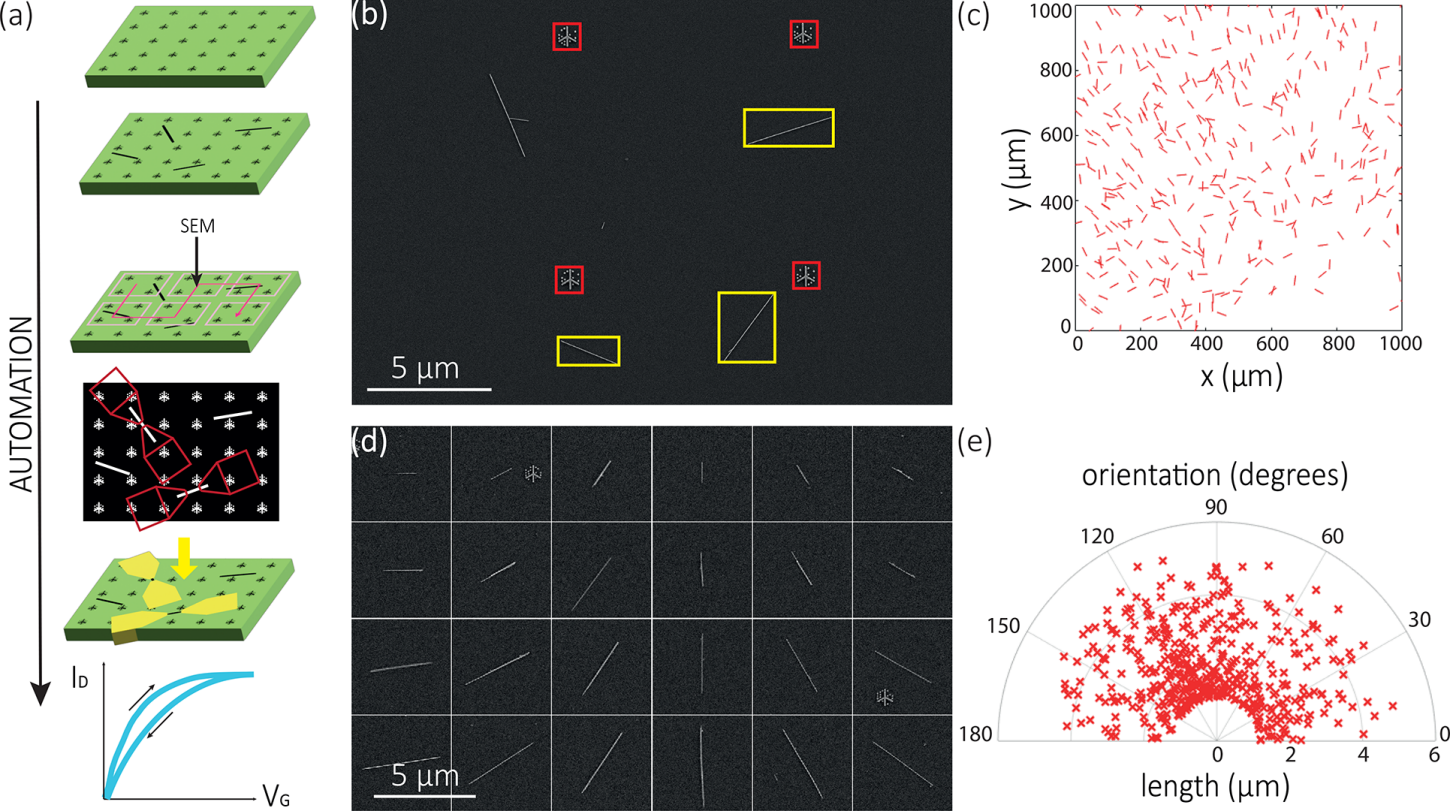
(a) Schematic diagram of the automated nanofabrication process: patterning LithoTags on a silicon chip, nanowire deposition, automated SEM imaging, CAD design, contact deposition, and measuring the fabricated device. (b) Annotated image of LithoTags (within red boxes) and successfully identified isolated InAs nanowires (within yellow boxes) on Si/SiO_2_ substrate. (c) Detected spatial distribution and orientation of isolated InAs nanowires in a 1 × 1 mm^2^ region of the map. Note that the graphical representations of nanowires in this image are enlarged for clarity and the apparent lengths are equal and uncorrelated with the measured lengths. (d) Demonstration of detection of isolated nanowires of different lengths and orientations. (e) Orientation distribution of detected isolated nanowires.

Figure 2a outlines our automated method of nanowire device fabrication. After patterning the substrate with LithoTags, depositing nanowires, and automated SEM imaging, we use a computer vision system to analyze SEM images and identify the nanowires we want to use for device fabrication. Our algorithm binarizes the image and then searches for features of interest based on area, circumference, length, width, aspect ratio, orientation, solidity, etc. In this case, these parameters are optimized to search for single nanowires by, for example, searching for features with a high aspect ratio. An example of nanowire detection from an arbitrary image from the SEM map is shown in Figure 2b. It can be seen that only isolated nanowires have been detected and the crossed nanowire pair in the top left is not selected. Figure 2c shows an example 1 × 1 mm^2^ area on a substrate with the position and orientation of all the isolated nanowires that were found by the algorithm. From this database of nanowires and their exact positions on the chip, we can systematically select nanowires with specific structural properties to study. For example, a set of isolated nanowires of specified lengths and orientations are identified from the substrate and shown in Figure 2d. Furthermore, the distribution of isolated nanowire orientations and lengths are shown in Figure 2e. Such structural and positional information is crucial for correlating with optical effects, such as nanowire lasing^16^ and polarization-dependent photoluminescence,^64^ and electronic effects, such as anisotropic spin–orbit interactions.^65^ A selection of 471 nanowires detected by the algorithm were inspected by hand, and of these all were found to correspond to actual nanowires. Of the nanowires detected, 11 corresponded to pairs of nanowires, and 6 nanowires overlapped with other features. This still gives a large number of nanowires suitable for device fabrication that have been identified in a fraction of the time compared to manual searching.

**Figure 3:**
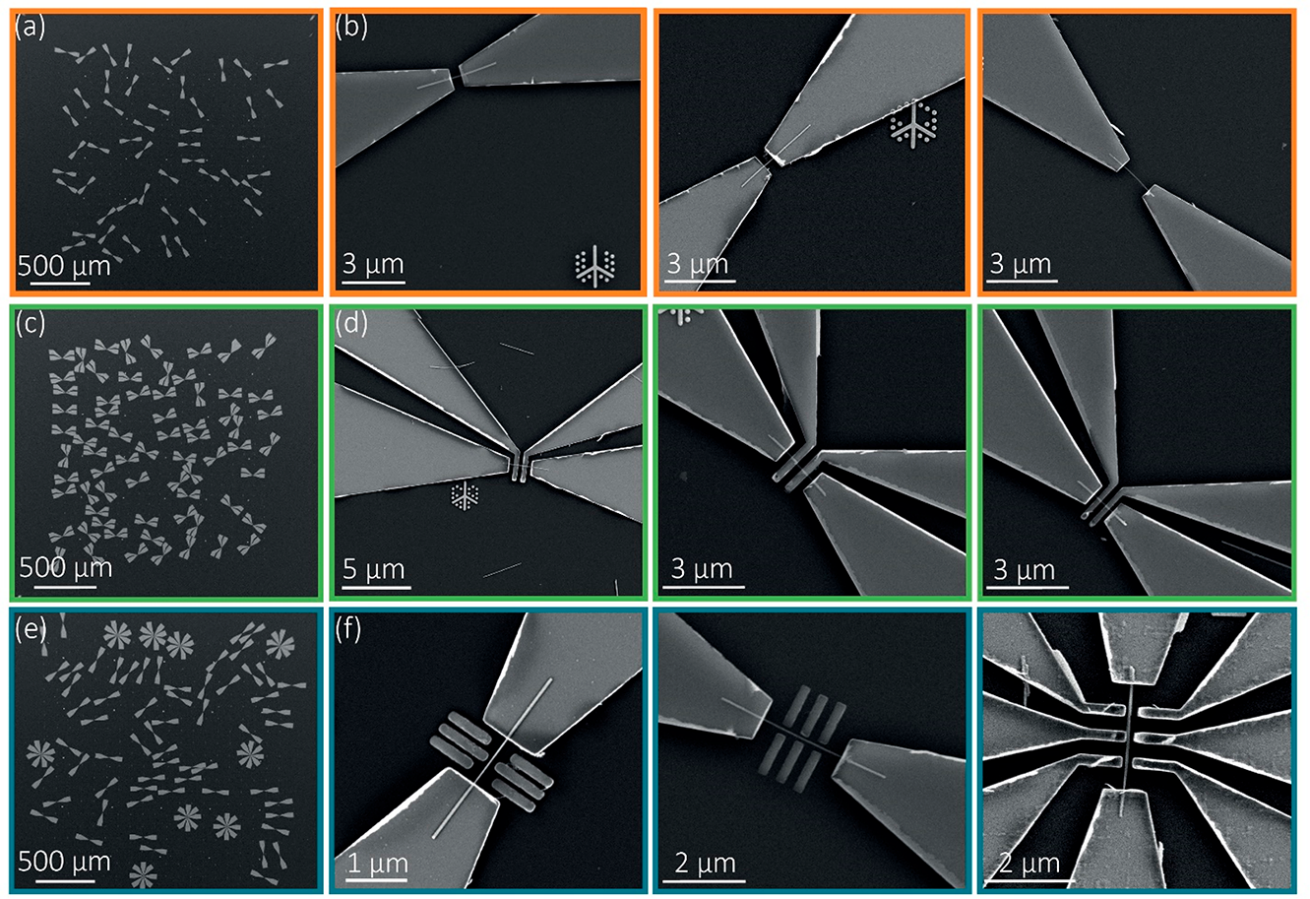
SEM image showing automatically fabricated (a) source and drain contacts with (b) higher magnification examples showing accurate alignment to the nanowire, (c) four-point contact with (d) higher magnification examples, and (e) double-quantum-dot gate with (f) higher magnification examples of nanowire devices on separate areas of a chip, each on a 2 × 2 mm^2^ area.

**Figure 4:**
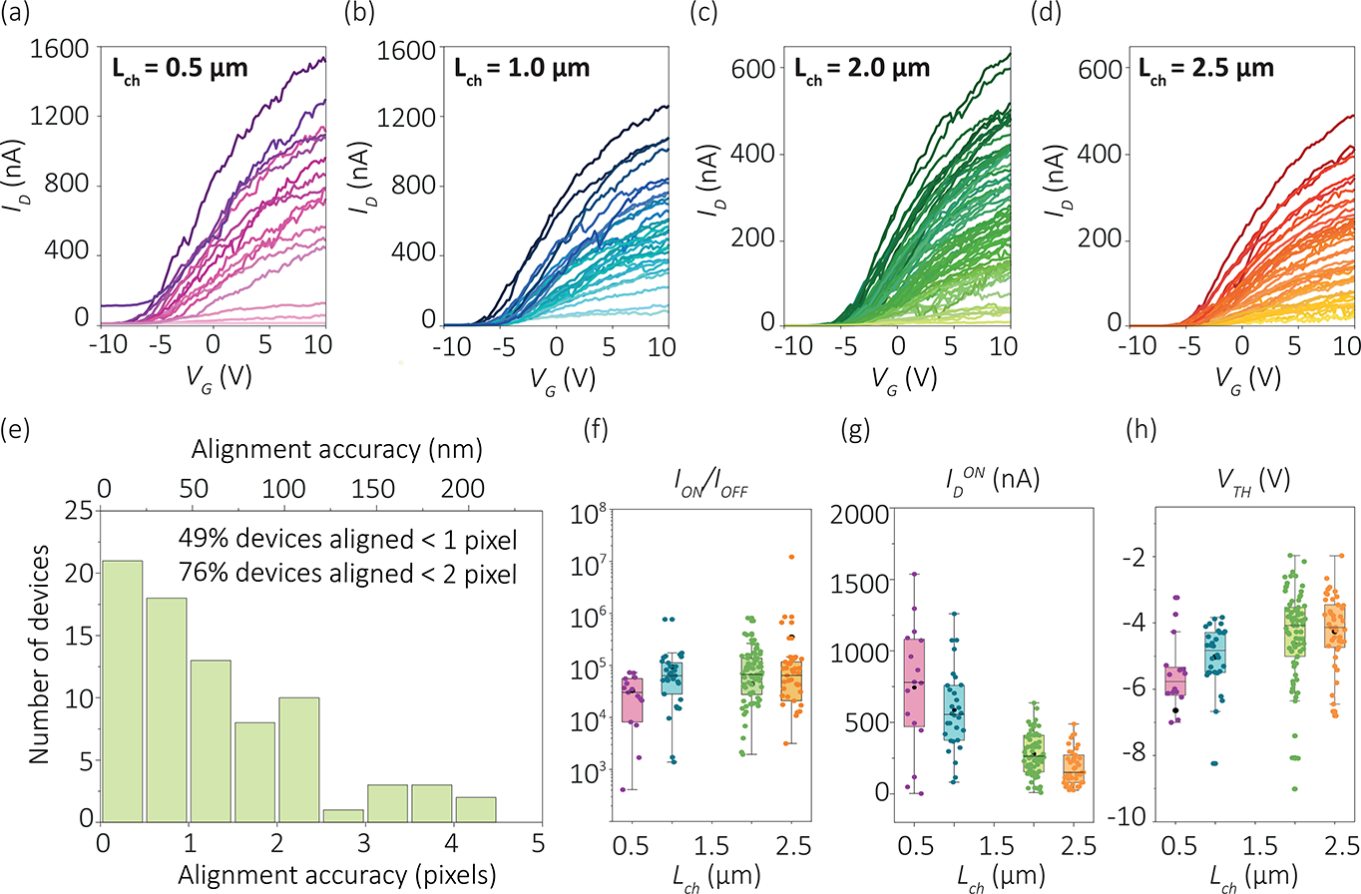
Transfer characteristics of automatically fabricated nanowire devices with (a) 0.5 μm channel length, (b) 1.0 μm channel length, (c) 2.0 μm channel length, and (d) 2.5 μm channel length at source–drain voltage *V*_DS_ = 10 mV. (e) Histogram of nanowire device misalignment measured from the center of the nanowire to the center of the electrode pattern. Statistical data of (f) on/off ratio, (g) peak current, and (h) threshold voltage measured in automatically fabricated nanowire devices.

Four additional regions (each 2 × 2 mm^2^) of nanowire location data were generated using automated high-throughput SEM mapping and LithoTag position detection. Each SEM image was 1024 pixels × 768 pixels, and the image area was 49 μm × 37 μm. To maximize the throughput of SEM imaging, a low image resolution was chosen. The 48 nm pixel size is larger than the nanowire diameter, representing an undersampled image. The results show that, despite this undersampling, the system enables high detection accuracy of both nanowires and LithoTags, reliable alignment of electrodes, and high device yield. After SEM imaging, we employed a custom pattern generation and alignment algorithm to automatically generate a CAD file of electrode patterns (Supporting Information Figure S6). This CAD file was then written on the nanowire chip by EBL, followed by development, ammonium sulfide etch, Ni deposition, and lift-off. We generated patterns for 267 nanowire devices on a chip, including 40 two-terminal devices (Figure 3a,b), 69 four-contact devices (Figure 3c,d), and 158 two-terminal devices with side gate structures (Figure 3e,f). The side gate structures are analogous to double-quantum-dot geometries used in various quantum devices,^66−69^ where the alignment accuracy determines the symmetry and efficiency of the capacitive coupling between the local gate and the nanowire. The as-designed separation between the side gate and the center of the nanowire is as low as 75 nm. The devices are designed with channel lengths *L*_ch_ from 0.5 to 2.5 μm, systematically generated by our code. Any desired pattern can be automatically generated and aligned, with tunable device dimensions. Channel length was chosen to match the length distribution of the nanowires, as shown in Figure 2e, and the bond pad dimensions of 40 × 40 μm^2^ were the optimal size for characterization in the probe station while also maintaining relatively compact device dimensions. To prevent contacts from overlapping, we can choose nanowires with specific orientations or include a minimum separation between the selected nanowires. After fabrication, high-resolution SEM images were taken of each nanowire to determine the accuracy of electrode alignment. Figure 3 panels a, c and e show arrays of devices fabricated over a large area of the substrate, and panels b, d, and f illustrate the corresponding higher magnification images exemplifying the accurate alignment to the isolated nanowires. Using the high-magnification images, we measured the relative alignment of the contacts with respect to the central axis of the nanowire. The electrode axis-to-nanowire axis alignment accuracy was within 2 pixels of the original microscope image in more than 76% of cases (Figure 4e). Potential sources of misalignment are the resolution of the original image data, where 1 pixel corresponds to 48 nm, the simplification and assumption of nanowires being straight in our pattern generation code, and the alignment accuracy of EBL, which is stated as ±8 nm for our system. In addition to this, development, metallization, and lift-off could result in broadening of the features. A subsequent set of devices were processed by the same procedure but using SEM images with twice the resolution (24 nm pixel size) for mapping. In this set, 71% of devices featured an electrode alignment accuracy within 2 pixels of the resolution of the original SEM images (Supporting Information Figure S7). The result indicates that the resolution of the original SEM image used for mapping is the main factor limiting electrode alignment accuracy at present. This limitation can be easily overcome by acquiring the original map at higher resolution. There is usually a compromise between mapping speed and resolution, but the advent of high-throughput microscopy techniques such as multi-beam SEM^70^ should circumvent this compromise.

Electrical characterization was performed on 267 automatically fabricated nanowire devices, with 202 nanowire devices demonstrating transistor switching behavior. Some devices showed damaged contacts due to processing issues during development, deposition, lift-off, or contamination. Regardless, 202 working nanowire devices still provide sufficient statistics for demonstration of automated alignment and fabrication. We measured the back-gate voltage (*V*_G_) transfer characteristics of nanowire devices with different channel lengths as shown in Figure 4a–d to compare the on/off ratio (*I*_ON_/*I*_OFF_), the peak current (*I*_D_^ON^), and the threshold voltage (*V*_TH_) (Figure 4f, g, h, respectively). It can be seen that *I*_ON_/*I*_OFF_ increases with increasing channel length from an average ∼10^4^ to ∼10^6^, and the average *I*_D_^ON^ decreases from ∼750 to ∼250 nA with increasing channel length. One potential source of discrepancies could be variations in the diameter (30 ± 5 nm), as it has been shown that mobility and threshold voltage are strongly diameter-dependent for InAs nanowires,^8^ in particular for those with diameters under 40 nm.^71^ The gate hysteresis remains approximately constant within error margins, and the average mobility is ∼1000 cm^2^/(V·s) for all channel lengths (Supporting Information Figure S8). All devices exhibit some hysteresis between forward and backward gate sweeps (Supporting Information Figure S9), indicating the presence of trap states, and all require positive gate voltage bias to “turn on” the conduction, indicating n-type behavior. We further investigate nanowire device functionality under time-dependent white-light illumination and find that the nanowires exhibit negative photoconductivity (Supporting Information Figure S10). These results demonstrate a systematic study of nanowire channel characteristics with statistically meaningful device numbers and show that the automated fabrication method is accurate and reliable enough for high-throughput nanowire device manufacturing.

## Conclusions

We demonstrate high-throughput automated InAs nanowire characterization and device fabrication achieved using a custom-made, lithography-optimized LithoTag fiducial marker system. We developed a computer vision algorithm to read LithoTags and detect isolated nanowires of specific length and orientation from microscopic images and automatically design aligned electrode patterns on selected nanowires to use for contact deposition. We fabricated over 200 nanowire devices on a single substrate and measured their electrical characteristics to demonstrate their functionality. Our results show that automatically aligned electrodes based on 30-nm-diameter nanowires were aligned to within 2 pixels, which can be further improved by using higher resolution SEM mapping. We extracted statistical data of the electronic and optoelectronic device characteristics from arrays of individual nanowire devices with systematically controlled device geometries. Our method enables high-throughput fabrication of nanomaterial devices, allows correlation between characterization techniques, and generates large nanomaterial property datasets, thereby saving hundreds of hours of researcher time spent on manual, repetitive tasks. The process is broadly applicable to other nanomaterial systems, as well as other imaging and nanofabrication techniques.

## Methods

### LithoTag

An implementation of the LithoTag design has been programmed in MATLAB. The code uses the MATLAB DXFLib to convert fiducial markers to DXF files for EBL patterning.^72^

### Fiducial Marker Fabrication

The markers were fabricated on oxidized Si (90 nm SiO_2_) chips spin-coated with PMMA 495 A8 at 4000 rpm for 45 s and baked for 3 min at 120 °C. The patterns were exposed using a Raith EBPG 5200 EBL system and developed in a 15:5:1 IPA:MIBK:MEK solution for 20 s. The markers were deposited by electron beam evaporation with Ti/Au (5/35 nm) at 0.1 Å/s, prior to lift-off in acetone.

### Nanowire Growth

Wurtzite InAs nanowires with an average diameter of 30 ± 5 nm, as measured by SEM for nanowires on the growth substrate, were grown by metal–organic chemical vapor deposition using Au nanoparticles as catalyst under the conditions described in ref (73).

### Nanowire Deposition

LithoTag-patterned substrates were first treated with O_2_ plasma to increase surface wettability. The as-grown nanowires on substrates were sonicated in IPA, then drop-cast onto the patterned chips and allowed to dry at room temperature, before being rinsed in IPA to remove residues.

### Device Fabrication

All devices were fabricated on 90 nm SiO_2_ on Si by spin-coating PMMA 495 A8 at 4000 rpm and exposing in a Raith EBPG 5200 EBL system. Nanowires were contacted with 60 nm of sputtered Ni, following an ammonium sulfide etch to improve the stability of the contacts.^49^ The etch was performed by leaving the developed sample in a 2% ammonium sulfide solution heated to 45 °C for 30 s, followed by rinsing with deionized water and air drying. After metallization, lift-off was performed in acetone, and the substrates were cleaned in IPA.

### Automated SEM Imaging

Large-scale imaging of InAs nanowires on LithoTag patterned substrates was performed using a Zeiss Gemini 300 SEM with a Python interface for controlled stage movement.

### Electrical Characterization

Electrical characterization of nanowire devices was performed using a probe station connected to a Keithley 4200-SCS semiconductor characterization system. The photoconductivity measurements were measured using a white-light illumination source. All measurements were carried out at room temperature under ambient conditions.

## Notes

Datasets related to this publication are available from the Cambridge University data repository at DOI: 10.17863/CAM.87937.

## Supporting Information Available

Fiducial marker designs, fiducial markers fabricated on silicon substrates of 2 × 2 μm size, fiducial markers fabricated on silicon substrates of 20 × 20 μm size, the LithoTag template, LithoTag detection in percentage as a function of Gaussian image blur, comparison of nanowire device CAD designs with SEM data, alignment accuracy of nanowire devices based on 24 nm pixel size SEM images, statistical analysis of forward and backward *V*_G_ sweeps of automatically fabricated devices of different channel lengths, and time-dependent negative photoconductivity in a nanowire device under white light illumination (PDF)

## References

[ref1] Ju L.; Shi Z.; Nair N.; Lv Y.; Jin C.; Velasco J.; Ojeda-Aristizabal C.; Bechtel H. A.; Martin M. C.; Zettl A.; Analytis J.; Wang F. Topological Valley Transport at Bilayer Graphene Domain Walls. Nature 2015, 520 (7549), 650–655. 10.1038/nature14364. 25901686

[ref2] Sangwan V. K.; Jariwala D.; Kim I. S.; Chen K.-S.; Marks T. J.; Lauhon L. J.; Hersam M. C. Gate-Tunable Memristive Phenomena Mediated by Grain Boundaries in Single-Layer MoS2. Nat. Nanotechnol. 2015, 10 (5), 403–406. 10.1038/nnano.2015.56. 25849785

[ref3] Wu W.; Zhang Q.; Zhou X.; Li L.; Su J.; Wang F.; Zhai T. Self-Powered Photovoltaic Photodetector Established on Lateral Monolayer MoS_2_-WS_2_ Heterostructures. Nano Energy 2018, 51, 45–53. 10.1016/j.nanoen.2018.06.049.

[ref4] Pezzini S.; Mišeikis V.; Piccinini G.; Forti S.; Pace S.; Engelke R.; Rossella F.; Watanabe K.; Taniguchi T.; Kim P.; Coletti C. 30°-Twisted Bilayer Graphene Quasicrystals from Chemical Vapor Deposition. Nano Lett. 2020, 20 (5), 3313–3319. 10.1021/acs.nanolett.0c00172. 32297749

[ref5] Stewart J. C.; Fan Y.; Danial J. S. H.; Goetz A.; Prasad A. S.; Burton O. J.; Alexander-Webber J. A.; Lee S. F.; Skoff S. M.; Babenko V.; Hofmann S. Quantum Emitter Localization in Layer-Engineered Hexagonal Boron Nitride. ACS Nano 2021, 15 (8), 13591–13603. 10.1021/acsnano.1c04467. 34347438

[ref6] Yang Z.; Albrow-Owen T.; Cui H.; Alexander-Webber J.; Gu F.; Wang X.; Wu T.-C.; Zhuge M.; Williams C.; Wang P.; Zayats A. V.; Cai W.; Dai L.; Hofmann S.; Overend M.; Tong L.; Yang Q.; Sun Z.; Hasan T. Single-Nanowire Spectrometers. Science 2019, 365 (6457), 1017–1020. 10.1126/science.aax8814. 31488686

[ref7] Smith L. W.; Batey J. O.; Alexander-Webber J. A.; Fan Y.; Hsieh Y.-C.; Fung S.-J.; Jevtics D.; Robertson J.; Guilhabert B. J. E.; Strain M. J.; Dawson M. D.; Hurtado A.; Griffiths J. P.; Beere H. E.; Jagadish C.; Burton O. J.; Hofmann S.; Chen T.-M.; Ritchie D. A.; Kelly M.; Joyce H. J.; Smith C. G. High-Throughput Electrical Characterization of Nanomaterials from Room to Cryogenic Temperatures. ACS Nano 2020, 14 (11), 15293–15305. 10.1021/acsnano.0c05622. 33104341

[ref8] Dayeh S. A.; Yu E. T.; Wang D. Transport Coefficients of InAs Nanowires as a Function of Diameter. Small 2009, 5 (1), 77–81. 10.1002/smll.200800969. 19040215

[ref9] Svensson J.; Dey A. W.; Jacobsson D.; Wernersson L.-E. III-V Nanowire Complementary Metal-Oxide Semiconductor Transistors Monolithically Integrated on Si. Nano Lett. 2015, 15 (12), 7898–7904. 10.1021/acs.nanolett.5b02936. 26595174

[ref10] Paladugu M.; Zou J.; Guo Y.; Zhang X.; Joyce H.; Gao Q.; Tan H.; Jagadish C.; Kim Y. Evolution of Wurtzite Structured GaAs Shells Around InAs Nanowire Cores. Nanoscale Res. Lett. 2009, 4 (8), 846 10.1007/s11671-009-9326-6. 20596432 PMC2893864

[ref11] Mourik V.; Zuo K.; Frolov S. M.; Plissard S. R.; Bakkers E. P. A. M.; Kouwenhoven L. P. Signatures of Majorana Fermions in Hybrid Superconductor-Semiconductor Nanowire Devices. Science 2012, 336 (6084), 1003–1007. 10.1126/science.1222360. 22499805

[ref12] Bryllert T.; Wernersson L.-E.; Froberg L. E.; Samuelson L. Vertical High-Mobility Wrap-Gated InAs Nanowire Transistor. IEEE Electron Device Lett. 2006, 27 (5), 323–325. 10.1109/LED.2006.873371.

[ref13] Ionescu A. M.; Riel H. Tunnel Field-Effect Transistors as Energy-Efficient Electronic Switches. Nature 2011, 479 (7373), 329–337. 10.1038/nature10679. 22094693

[ref14] Wong-Leung J.; Yang I.; Li Z.; Karuturi S. K.; Fu L.; Tan H. H.; Jagadish C. Engineering III-V Semiconductor Nanowires for Device Applications. Adv. Mater. 2020, 32 (18), 1904359 10.1002/adma.201904359. 31621966

[ref15] Storm K.; Halvardsson F.; Heurlin M.; Lindgren D.; Gustafsson A.; Wu P. M.; Monemar B.; Samuelson L. Spatially Resolved Hall Effect Measurement in a Single Semiconductor Nanowire. Nat. Nanotechnol. 2012, 7 (11), 718–722. 10.1038/nnano.2012.190. 23103932

[ref16] Alanis J. A.; Saxena D.; Mokkapati S.; Jiang N.; Peng K.; Tang X.; Fu L.; Tan H. H.; Jagadish C.; Parkinson P. Large-Scale Statistics for Threshold Optimization of Optically Pumped Nanowire Lasers. Nano Lett. 2017, 17 (8), 4860–4865. 10.1021/acs.nanolett.7b01725. 28732157

[ref17] Parkinson P.; Alanis J. A.; Peng K.; Saxena D.; Mokkapati S.; Jiang N.; Fu L.; Tan H. H.; Jagadish C. Modal Refractive Index Measurement in Nanowire Lasers—a Correlative Approach. Nano Futur. 2018, 2 (3), 035004 10.1088/2399-1984/aad0c6.

[ref18] Bai H.; Wu S. Deep-Learning-Based Nanowire Detection in AFM Images for Automated Nanomanipulation. Nanotechnol. Precis. Eng. 2021, 4 (1), 013002 10.1063/10.0003218.

[ref19] Jevtics D.; McPhillimy J.; Guilhabert B.; Alanis J. A.; Tan H. H.; Jagadish C.; Dawson M. D.; Hurtado A.; Parkinson P.; Strain M. J. Characterization, Selection, and Microassembly of Nanowire Laser Systems. Nano Lett. 2020, 20 (3), 1862–1868. 10.1021/acs.nanolett.9b05078. 32017573 PMC7146854

[ref20] Ye X.; Zhang Y.; Ru C.; Luo J.; Xie S.; Sun Y. Automated Pick-Place of Silicon Nanowires. IEEE Trans. Autom. Sci. Eng. 2013, 10 (3), 554–561. 10.1109/TASE.2013.2244082.

[ref21] Blanc P.; Heiss M.; Colombo C.; Mallorquì A. D.; Safaei T. S.; Krogstrup P.; Nygård J.; Morral A. F. i. Electrical Contacts to Single Nanowires: A Scalable Method Allowing Multiple Devices on a Chip. Application to a Single Nanowire Radial p-i-n Junction. Int. J. Nanotechnol. 2013, 10 (5–7), 419 10.1504/IJNT.2013.053513.

[ref22] Kalinin S. V.; Ziatdinov M.; Hinkle J.; Jesse S.; Ghosh A.; Kelley K. P.; Lupini A. R.; Sumpter B. G.; Vasudevan R. K. Automated and Autonomous Experiments in Electron and Scanning Probe Microscopy. ACS Nano 2021, 15 (8), 12604–12627. 10.1021/acsnano.1c02104. 34269558

[ref23] Rivenson Y.; Göröcs Z.; Günaydin H.; Zhang Y.; Wang H.; Ozcan A. Deep Learning Microscopy. Optica 2017, 4 (11), 1437 10.1364/OPTICA.4.001437.

[ref24] von Chamier L.; Laine R. F.; Jukkala J.; Spahn C.; Krentzel D.; Nehme E.; Lerche M.; Hernández-Pérez S.; Mattila P. K.; Karinou E.; Holden S.; Solak A. C.; Krull A.; Buchholz T.-O.; Jones M. L.; Royer L. A.; Leterrier C.; Shechtman Y.; Jug F.; Heilemann M.; Jacquemet G.; Henriques R. Democratising Deep Learning for Microscopy with ZeroCostDL4Mic. Nat. Commun. 2021, 12 (1), 2276 10.1038/s41467-021-22518-0. 33859193 PMC8050272

[ref25] Midtvedt B.; Helgadottir S.; Argun A.; Pineda J.; Midtvedt D.; Volpe G. Quantitative Digital Microscopy with Deep Learning. Appl. Phys. Rev. 2021, 8 (1), 011310 10.1063/5.0034891.

[ref26] Han B.; Lin Y.; Yang Y.; Mao N.; Li W.; Wang H.; Yasuda K.; Wang X.; Fatemi V.; Zhou L.; Wang J. I. -Ja.; Ma Q.; Cao Y.; Rodan-Legrain D.; Bie Y.; Navarro-Moratalla E.; Klein D.; MacNeill D.; Wu S.; Kitadai H.; Ling X.; Jarillo-Herrero P.; Kong J.; Yin J.; Palacios T. Deep-Learning-Enabled Fast Optical Identification and Characterization of 2D Materials. Adv. Mater. 2020, 32 (29), 2000953 10.1002/adma.202000953. 32519397

[ref27] Yang J.; Yao H. Automated Identification and Characterization of Two-Dimensional Materials via Machine Learning-Based Processing of Optical Microscope Images. Extrem. Mech. Lett. 2020, 39, 100771 10.1016/j.eml.2020.100771.

[ref28] Cellini F.; Lavini F.; Berger C.; de Heer W.; Riedo E. Layer Dependence of Graphene-Diamene Phase Transition in Epitaxial and Exfoliated Few-Layer Graphene Using Machine Learning. 2D Mater. 2019, 6 (3), 035043 10.1088/2053-1583/ab1b9f.

[ref29] Lin X.; Si Z.; Fu W.; Yang J.; Guo S.; Cao Y.; Zhang J.; Wang X.; Liu P.; Jiang K.; Zhao W. Intelligent Identification of Two-Dimensional Nanostructures by Machine-Learning Optical Microscopy. Nano Res. 2018, 11 (12), 6316–6324. 10.1007/s12274-018-2155-0.

[ref30] Schorb M.; Haberbosch I.; Hagen W. J. H.; Schwab Y.; Mastronarde D. N. Software Tools for Automated Transmission Electron Microscopy. Nat. Methods 2019, 16 (6), 471–477. 10.1038/s41592-019-0396-9. 31086343 PMC7000238

[ref31] Carter A. R.; King G. M.; Ulrich T. A.; Halsey W.; Alchenberger D.; Perkins T. T. Stabilization of an Optical Microscope to 01 Nm in Three Dimensions. Appl. Opt. 2007, 46 (3), 421 10.1364/AO.46.000421. 17228390

[ref32] Acher O.; Nguyen T. L. Turning a Machine Vision Camera into a High Precision Position and Angle Encoder: NanoGPS-OxyO. Proc SPIE: Optical Measurement Systems for Industrial Inspection XI 2019, 11056, 685–692. 10.1117/12.2524938.

[ref33] Acher O.; Nguyên T.-L.; Podzorov A.; Leroy M.; Carles P.-A.; Legendre S. An Efficient Solution for Correlative Microscopy and Co-Localized Observations Based on Multiscale Multimodal Machine-Readable NanoGPS Tags. Meas. Sci. Technol. 2021, 32 (4), 045402 10.1088/1361-6501/abce39.

[ref34] Peng K.; Jevtics D.; Zhang F.; Sterzl S.; Damry D. A.; Rothmann M. U.; Guilhabert B.; Strain M. J.; Tan H. H.; Herz L. M.; Fu L.; Dawson M. D.; Hurtado A.; Jagadish C.; Johnston M. B. Three-Dimensional Cross-Nanowire Networks Recover Full Terahertz State. Science 2020, 368 (6490), 510–513. 10.1126/science.abb0924. 32355027

[ref35] Xu W.-Z.; Ren F.-F.; Jevtics D.; Hurtado A.; Li L.; Gao Q.; Ye J.; Wang F.; Guilhabert B.; Fu L.; Lu H.; Zhang R.; Tan H. H.; Dawson M. D.; Jagadish C. Vertically Emitting Indium Phosphide Nanowire Lasers. Nano Lett. 2018, 18 (6), 3414–3420. 10.1021/acs.nanolett.8b00334. 29781625

[ref36] Fiala M. ARTag, a Fiducial Marker System Using Digital Techniques. 2005 IEEE Computer Society Conference on Computer Vision and Pattern Recognition (CVPR’05) 2005, 2, 590–596. 10.1109/CVPR.2005.74.

[ref37] Bergamasco F.; Albarelli A.; Cosmo L.; Rodola E.; Torsello A. An Accurate and Robust Artificial Marker Based on Cyclic Codes. IEEE Trans. Pattern Anal. Mach. Intell. 2016, 38 (12), 2359–2373. 10.1109/TPAMI.2016.2519024. 26800529

[ref38] Wang J.; Olson E. AprilTag 2: Efficient and Robust Fiducial Detection. 2016 IEEE/RSJ International Conference on Intelligent Robots and Systems (IROS) 2016, 4193–4198. 10.1109/IROS.2016.7759617.

[ref39] Chang T. H. P. Proximity Effect in Electron-beam Lithography. J. Vac. Sci. Technol. 1975, 12 (6), 1271–1275. 10.1116/1.568515.

[ref40] Yoon G.; Kim I.; So S.; Mun J.; Kim M.; Rho J. Fabrication of Three-Dimensional Suspended, Interlayered and Hierarchical Nanostructures by Accuracy-Improved Electron Beam Lithography Overlay. Sci. Rep. 2017, 7 (1), 6668 10.1038/s41598-017-06833-5. 28751643 PMC5532261

[ref41] Cui Z. Nanofabrication: principles, capabilities and limits; Springer, New York, NY, 2016. 10.1007/978-3-319-39361-2.

[ref42] Howard R. E.; Prober D. E. Nanometer-Scale Fabrication Techniques. VLSI Electronics Microstructure Science 1982, 5, 145–189. 10.1016/b978-0-12-234105-2.50009-4.

[ref43] Mack C. A. Fundamental Principles of Optical Lithography: The Science of Microfabrication; John Wiley and Sons: Chichester, 2007.

[ref44] Sklar B. Digital Communications: Fundamentals and Applications; Prentice-Hall, Inc.: Upper Saddle River, NJ, 1998.

[ref45] Wicker S. B. Error Control Systems for Digital Communication and Storage; Prentice-Hall, Inc.: Upper Saddle River, NJ, 1995.

[ref46] Dayeh S. A.; Aplin D. P. R.; Zhou X.; Yu P. K. L.; Yu E. T.; Wang D. High Electron Mobility InAs Nanowire Field-Effect Transistors. Small 2007, 3 (2), 326–332. 10.1002/smll.200600379. 17199246

[ref47] Riel H.; Wernersson L. E.; Hong M.; Del Alamo J. A. III-V Compound Semiconductor Transistors - From Planar to Nanowire Structures. MRS Bull. 2014, 39 (8), 668–677. 10.1557/mrs.2014.137.

[ref48] Olsson L. Ö.; Andersson C. B. M.; Håkansson M. C.; Kanski J.; Ilver L.; Karlsson U. O. Charge Accumulation at InAs Surfaces. Phys. Rev. Lett. 1996, 76 (19), 3626–3629. 10.1103/PhysRevLett.76.3626. 10061015

[ref49] Suyatin D. B.; Thelander C.; Björk M. T.; Maximov I.; Samuelson L. Sulfur Passivation for Ohmic Contact Formation to InAs Nanowires. Nanotechnology 2007, 18 (10), 105307 10.1088/0957-4484/18/10/105307.

[ref50] Guo N.; Hu W.; Liao L.; Yip S.; Ho J. C.; Miao J.; Zhang Z.; Zou J.; Jiang T.; Wu S.; Chen X.; Lu W. Anomalous and Highly Efficient InAs Nanowire Phototransistors Based on Majority Carrier Transport at Room Temperature. Adv. Mater. 2014, 26 (48), 8203–8209. 10.1002/adma.201403664. 25352322

[ref51] Li J.; Yan X.; Sun F.; Zhang X.; Ren X. Anomalous Photoconductive Behavior of a Single InAs Nanowire Photodetector. Appl. Phys. Lett. 2015, 107 (26), 263103 10.1063/1.4938752.

[ref52] Han Y.; Zheng X.; Fu M.; Pan D.; Li X.; Guo Y.; Zhao J.; Chen Q. Negative Photoconductivity of InAs Nanowires. Phys. Chem. Chem. Phys. 2016, 18 (2), 818–826. 10.1039/C5CP06139C. 26631367

[ref53] Yang Y.; Peng X.; Kim H.-S.; Kim T.; Jeon S.; Kang H. K.; Choi W.; Song J.; Doh Y.-J.; Yu D. Hot Carrier Trapping Induced Negative Photoconductance in InAs Nanowires toward Novel Nonvolatile Memory. Nano Lett. 2015, 15 (9), 5875–5882. 10.1021/acs.nanolett.5b01962. 26226506

[ref54] Fang H.; Hu W.; Wang P.; Guo N.; Luo W.; Zheng D.; Gong F.; Luo M.; Tian H.; Zhang X.; Luo C.; Wu X.; Chen P.; Liao L.; Pan A.; Chen X.; Lu W. Visible Light-Assisted High-Performance Mid-Infrared Photodetectors Based on Single InAs Nanowire. Nano Lett. 2016, 16 (10), 6416–6424. 10.1021/acs.nanolett.6b02860. 27598791

[ref55] Alexander-Webber J. A.; Groschner C. K.; Sagade A. A.; Tainter G.; Gonzalez-Zalba M. F.; Di Pietro R.; Wong-Leung J.; Tan H. H.; Jagadish C.; Hofmann S.; Joyce H. J. Engineering the Photoresponse of InAs Nanowires. ACS Appl. Mater. Interfaces 2017, 9 (50), 43993–44000. 10.1021/acsami.7b14415. 29171260

[ref56] Miao J.; Hu W.; Guo N.; Lu Z.; Zou X.; Liao L.; Shi S.; Chen P.; Fan Z.; Ho J. C.; Li T.-X.; Chen X. S.; Lu W. Single InAs Nanowire Room-Temperature Near-Infrared Photodetectors. ACS Nano 2014, 8 (4), 3628–3635. 10.1021/nn500201g. 24592971

[ref57] Lapierre R. R.; Robson M.; Azizur-Rahman K. M.; Kuyanov P. A Review of III-V Nanowire Infrared Photodetectors and Sensors. J. Phys. D. Appl. Phys. 2017, 50 (12), 123001 10.1088/1361-6463/aa5ab3.

[ref58] Das A.; Ronen Y.; Most Y.; Oreg Y.; Heiblum M.; Shtrikman H. Zero-Bias Peaks and Splitting in an Al-InAs Nanowire Topological Superconductor as a Signature of Majorana Fermions. Nat. Phys. 2012, 8 (12), 887–895. 10.1038/nphys2479.

[ref59] Frolov S. M.; Manfra M. J.; Sau J. D. Topological Superconductivity in Hybrid Devices. Nat. Phys. 2020, 16 (7), 718–724. 10.1038/s41567-020-0925-6.

[ref60] Liu Z.; Luo T.; Liang B.; Chen G.; Yu G.; Xie X.; Chen D.; Shen G. High-Detectivity InAs Nanowire Photodetectors with Spectral Response from Ultraviolet to near-Infrared. Nano Res. 2013, 6 (11), 775–783. 10.1007/s12274-013-0356-0.

[ref61] Wei W.; Bao X.-Y.; Soci C.; Ding Y.; Wang Z.-L.; Wang D. Direct Heteroepitaxy of Vertical InAs Nanowires on Si Substrates for Broad Band Photovoltaics and Photodetection. Nano Lett. 2009, 9 (8), 2926–2934. 10.1021/nl901270n. 19624100

[ref62] Mallorquí A. D.; Alarcón-Lladó E.; Russo-Averchi E.; Tütüncüoglu G.; Matteini F.; Rüffer D.; Morral A. F. i. Characterization and Analysis of InA s/p -Si Heterojunction Nanowire-Based Solar Cell. J. Phys. D. Appl. Phys. 2014, 47 (39), 394017 10.1088/0022-3727/47/39/394017.

[ref63] Li B.; Wei W.; Yan X.; Zhang X.; Liu P.; Luo Y.; Zheng J.; Lu Q.; Lin Q.; Ren X. Mimicking Synaptic Functionality with an InAs Nanowire Phototransistor. Nanotechnology 2018, 29 (46), 464004 10.1088/1361-6528/aadf63. 30246691

[ref64] Mishra A.; Titova L. V.; Hoang T. B.; Jackson H. E.; Smith L. M.; Yarrison-Rice J. M.; Kim Y.; Joyce H. J.; Gao Q.; Tan H. H.; Jagadish C. Polarization and Temperature Dependence of Photoluminescence from Zincblende and Wurtzite InP Nanowires. Appl. Phys. Lett. 2007, 91 (26), 263104 10.1063/1.2828034.

[ref65] Iorio A.; Rocci M.; Bours L.; Carrega M.; Zannier V.; Sorba L.; Roddaro S.; Giazotto F.; Strambini E. Vectorial Control of the Spin-Orbit Interaction in Suspended InAs Nanowires. Nano Lett. 2019, 19 (2), 652–657. 10.1021/acs.nanolett.8b02828. 30398889

[ref66] Bordoloi A.; Zannier V.; Sorba L.; Schönenberger C.; Baumgartner A. A Double Quantum Dot Spin Valve. Commun. Phys. 2020, 3 (1), 135 10.1038/s42005-020-00405-2.

[ref67] Fasth C.; Fuhrer A.; Samuelson L. Quantum Dots Defined in InAs Quantum Wires by Local Gate Electrodes. AIP Conference Proceedings 2007, 893, 843–844. 10.1063/1.2730154.

[ref68] Nilsson M.; Chen I.-J.; Lehmann S.; Maulerova V.; Dick K. A.; Thelander C. Parallel-Coupled Quantum Dots in InAs Nanowires. Nano Lett. 2017, 17 (12), 7847–7852. 10.1021/acs.nanolett.7b04090. 29172541

[ref69] Baba S.; Matsuo S.; Kamata H.; Deacon R. S.; Oiwa A.; Li K.; Jeppesen S.; Samuelson L.; Xu H. Q.; Tarucha S. Gate Tunable Parallel Double Quantum Dots in InAs Double-Nanowire Devices. Appl. Phys. Lett. 2017, 111 (23), 233513 10.1063/1.4997646.

[ref70] Eberle A. L.; Mikula S.; Schalek R.; Lichtman J.; Tate M. L. K.; Zeidler D. High-Resolution, High-Throughput Imaging with a Multibeam Scanning Electron Microscope. J. Microsc. 2015, 259 (2), 114–120. 10.1111/jmi.12224. 25627873 PMC4670696

[ref71] Scheffler M.; Nadj-Perge S.; Kouwenhoven L. P.; Borgström M. T.; Bakkers E. P. A. M. Diameter-Dependent Conductance of InAs Nanowires. J. Appl. Phys. 2009, 106 (12), 124303 10.1063/1.3270259.

[ref72] Kwiatek G. DXFLib, version 1.2.0.0; MathWorks: MATLAB Central File Exchange, 2021.

[ref73] Joyce H. J.; Wong-Leung J.; Gao Q.; Tan H. H.; Jagadish C. Phase Perfection in Zinc Blende and Wurtzite III-V Nanowires Using Basic Growth Parameters. Nano Lett. 2010, 10 (3), 908–915. 10.1021/nl903688v. 20131909

