# Supplementary material for: Automated Computer Vision-Enabled Manufacturing of Nanowire Devices
Source: ACS Nano. 2022 Sep 26;16(11):18009–17. doi: 10.1021/acsnano.2c08187 (PMC9706672; doi:10.1021/acsnano.2c08187)
Supplement: Supplementary file 1 — nn2c08187_si_001.pdf [file nn2c08187_si_001.pdf]

SUPPLEMENTAL INFORMATION

Automated computer vision-enabled manufacturing of nanowire devices

*Teja Potočnik[1] *, Peter J. Christopher[1], Ralf Mouthaan[1], Thomas Albrow-Owen[1], Oliver J. Burton[1], Chennupati Jagadish[2], Hark Hoe Tan[2], Timothy D. Wilkinson[1], Stephan Hofmann[1], Hannah J. Joyce[1], Jack A. Alexander-Webber[1] **

[1]Department of Engineering, University of Cambridge, 9 JJ Thompson Avenue, Cambridge CB3 0FA, United Kingdom

[2]Australian Research Council Centre of Excellence for Transformative Meta-Optical Systems, Department of Electronic Materials Engineering, Research School of Physics and Engineering, The Australian National University, Canberra, ACT 2600, Australia

To demonstrate the significance of LithoTag design we compare our system to existing fiducial markers. There are many different fiducial marker systems, and their designs depend heavily on their application. **Figure S1** shows some of the more common fiducial markers. One of the first fiducial marker designs used a binary concentric contrasting circles design,[1] which

was later improved by including data rings[2] and colors in combination with position detection,[3,4] thus significantly increasing the information density. Perhaps the most common is the 2D barcode system QR code, where the outer three corners provide information about the orientation of the marker, and the inside of the marker contains a significant information payload.[5] The other most common fiducial markers are ARToolKit[6] and ARTag, which use a binary interior system to convey information.[7] However, the drawbacks of these systems are high false-positive detection rates[7] that increase with reducing marker size and are limited by the resolution of the imaging system, and image contrast. This problem has been addressed with further developments in fiducial marker designs, namely the CalTag system, which uses checker patterns for accurate subpixel calibration,[8] the AprilTag system, which uses lexicographic codes to demonstrate an improvement in detection of markers with reduced size,[9] and the RuneTag system, which uses patterns of circular dots of different sizes,[10] resulting in significantly improved detection rates. These tags have been designed for the applications that are visible with a naked eye, but the challenge of encoding and detecting location information on a sub-micron scale has not been addressed.

To demonstrate the challenges associated with fiducial marker patterning when approaching the lithography feature resolution limit we pattern a set of the most common fiducial markers.

Markers were fabricated using electron beam lithography (EBL) on an oxidized silicon substrate with and metallized with sputtered W followed by lift-off. **Figure S2** shows SEM images of EBL patterned CCC, ARToolKit, ARTag, CalTag, AprilTag and RuneTag with a marker size of ~2x2 μm^2, and **Figure S3** with a marker size of ~20x20 μm^2. It is clear that not all features of the fiducial markers are appropriate for nanofabrication techniques, as their pattern fidelity is not maintained during processing. ARToolKit, ARTag and AprilTag show damaged edges, which makes them unsuitable for the use in such small scale applications, since they rely on edge detection. The CCC and RuneTag are completely destroyed during lift-off, and also show bridging between features as a result of proximity effects or higher density of exposed features. **Figure S2** shows non-lifted areas due to some exposed regions being enclosed, and the corners appear to be rounded due to a combination of effects, such as resolution of the resist and electron beam, proximity effects and minimum grain size of the sputtered W. Our system LithoTag has been designed to overcome these issues. Considering the minimum resolution of the lithography system, the smallest features of the marker must be larger than the minimum feature resolution. With the circle diameter being the limiting size factor, the smallest circle size for the LithoTag that can be achieved is about the same as the

minimum feature resolution. The same minimum circle size in a RuneTag would mean the whole marker is almost twice the size of the LithoTag.

Previous fiducial markers such as AprilTag, ARToolKit and CalTag have been designed to retain detection accuracy for changes in projection angles, as they use sharp line edges and corners for detection. The LithoTag detection system does not use sharp edges as they are more likely to be damaged during processing. Instead, a convolutional technique is used for detection, which is not very robust to changes in viewing projection angle and so the marker can only ensure detection reliability if viewed from approximately above. However, for the purposes of nano-fabrication, the changes in projection angle viewing are a favorable trade-off against features such as oversaturation resistance and minimum resolution, where LithoTag has significant advantages.

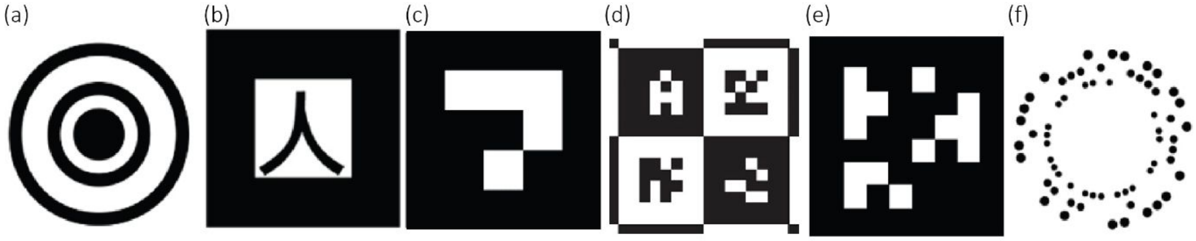

Figure S1: (a) Concentric Contrastic Circles, (b) ARToolKit, (c) ARTag, (d) CalTag, (e) AprilTag and (f) RuneTag fiducial marker.

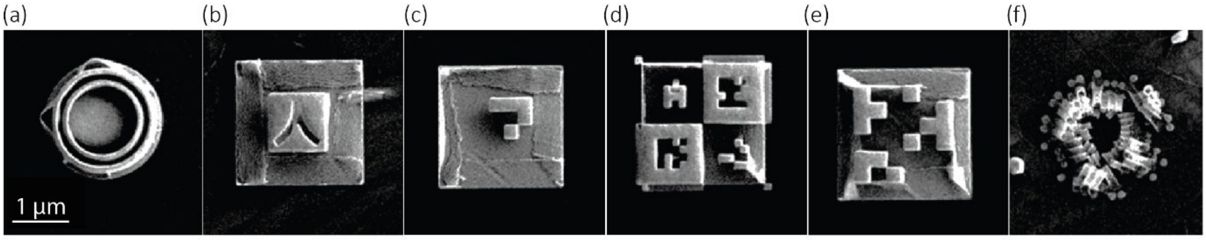

Figure S2: (a) Concentric Contrastic Circles, (b) ARToolKit, (c) ARTag, (d) CalTag, (e) AprilTag and (f) RuneTag fiducial marker fabricated on silicon substrate with Ni sputtering.

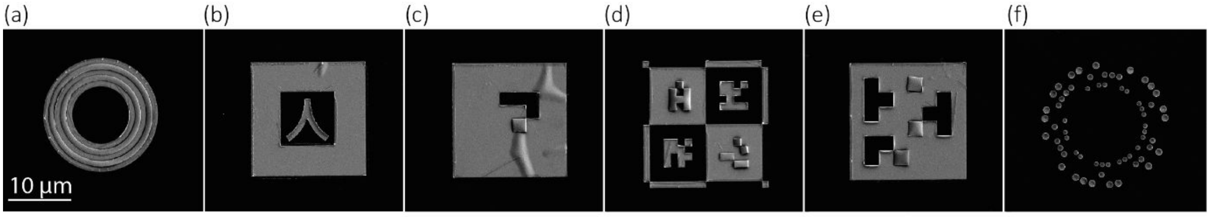

Figure S3: (a) Concentric Contrastic Circles, (b) ARToolKit, (c) ARTag, (d) CalTag, (e) AprilTag and (f) RuneTag fiducial marker fabricated on silicon substrate with Ni sputtering.

Figure S4 shows the LithoTag template used for LithoTag detection from SEM images. The detection accuracy as a function of Gaussian blur is shown in **Figure S5**, where the detected tags denote the markers being detected from the background, and the recognized tags refer to the markers with correctly identified coordinates.

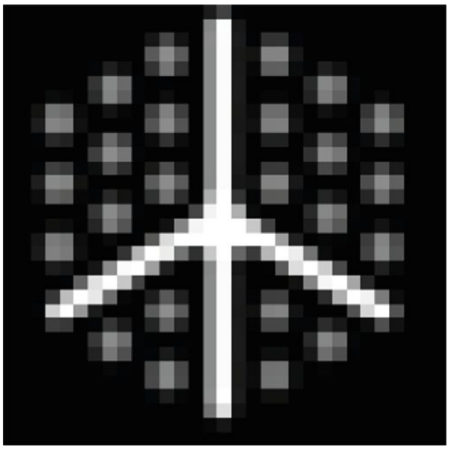

Figure S4: LithoTag template used for convolution detection method.

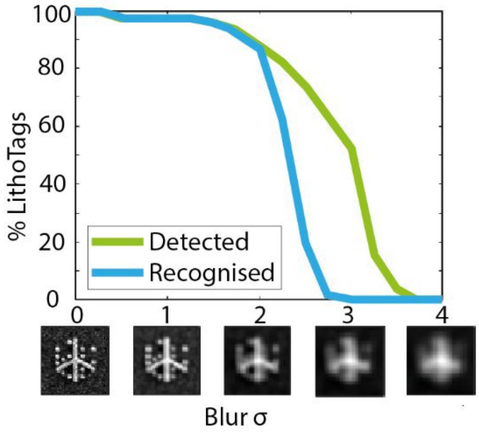

Figure S5: LithoTag detection as a function of Gaussian blur.

Figure S6a shows the output CAD file that has been generated by computer-vision algorithm after obtaining the information on isolated nanowire locations, with the contacts being drawn with respect to each nanowire centre and rotated along its orientation direction. **Figure S6**b shows an SEM image taken of the same area after deposition of the contacts to show the automated fabrication accuracy.

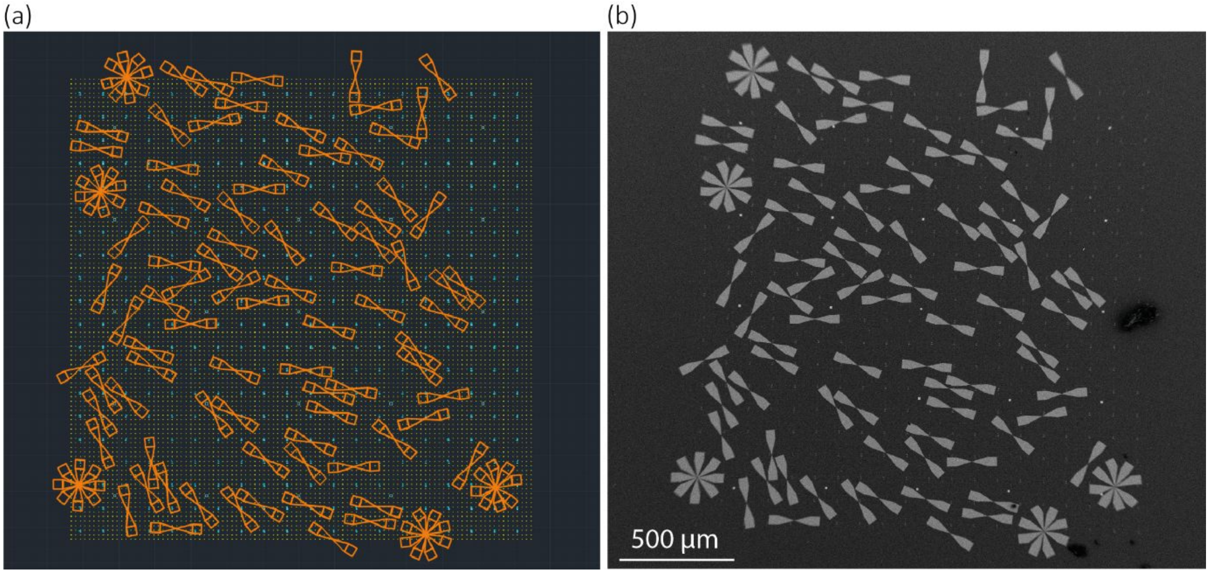

Figure S6: (a) Output CAD design as generated by the computer-vision and electrode pattern generation algorithm and (b) SEM image of automatically fabricated nanowire devices on silicon substrate of the same region.

Figure S7 shows the measured alignment of automatically fabricated nanowire devices. It was measured from SEM images after fabrication of the contacts, as the relative alignment between the central axis of the nanowire to the contact electrode axis.

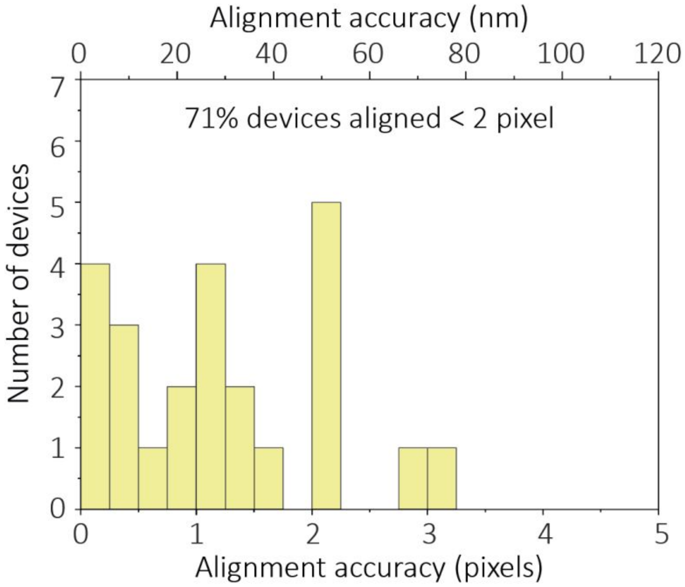

Figure S7: Alignment accuracy of automatically fabricated nanowire devices.

The statistical data obtained from 202 automatically fabricated nanowire devices as a function of channel length is shown in **Figure S8**, showing the field effect mobility and hysteresis obtained from transfer measurements. Hysteresis here is defined as the difference in threshold voltage from the up sweep and down sweep.

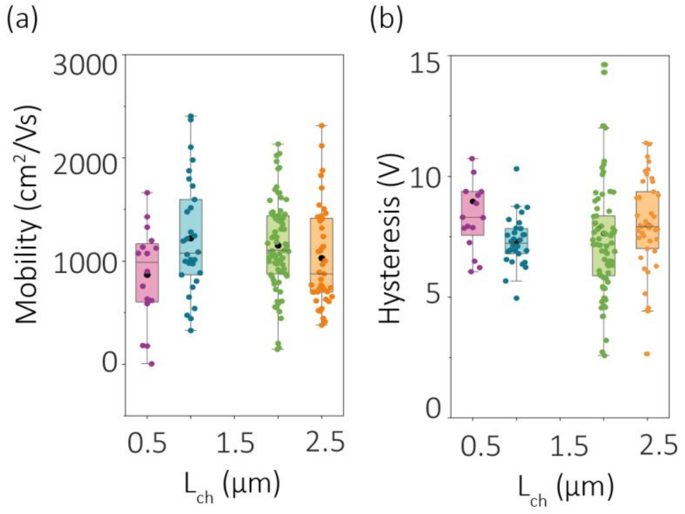

Figure S8: Statistical data of (a) mobility and (b) hysteresis measured in automatically fabricated nanowire devices.

Transfer characteristics measurement of automatically fabricated nanowire device is shown in **Figure S9**, with the up sweep and down sweep showing hysteresis behaviour, which was observed for all 202 devices measured.

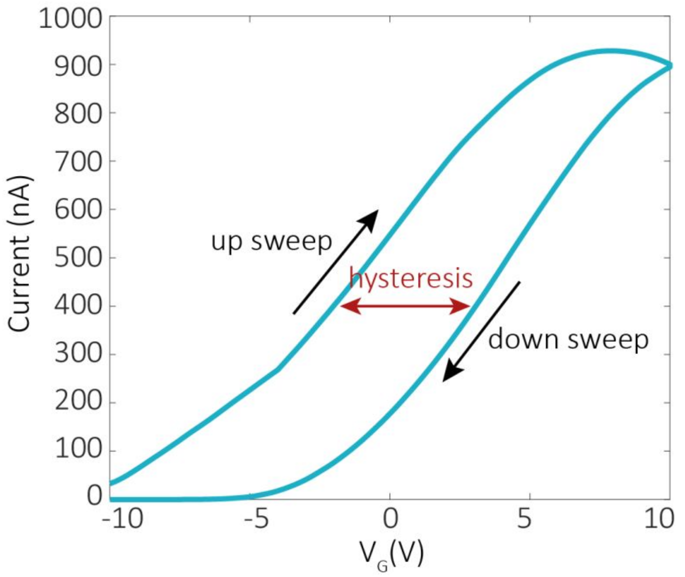

Figure S9: Transfer characteristics of automatically fabricated nanowire device showing hysteresis behaviour between forward and backward sweeps.

The transient photoresponse measures the current with respect to applied back gate voltage of 0 V and source-drain bias of 1 V (**Figure S10**). For an individual nanowire device with 1 μm channel length, the drain current drops by an order of magnitude after illumination, which can be predominantly attributed to InAs surface states.[11] Highly mobile electrons contribute to conduction along the channel in dark condition. Once the device is illuminated, electron-hole pairs are generated and the electrons can become trapped in the surface states in the native oxide, thus not contributing to the conduction. The photoexcited holes are left to recombine with equilibrium electrons, further reducing the number of free electrons in the channel and contributing to negative photoconductivity. Turning the light off again, the current gradually

increases to approximately 60% of the dark current within 10 seconds. We demonstrate multiple NPC cycles by turning the light source on and off every 10 seconds for 100 seconds. It has been shown that the current would be expected to reach the original dark current magnitude after turning the light source off given enough time for recovery.[12,13]

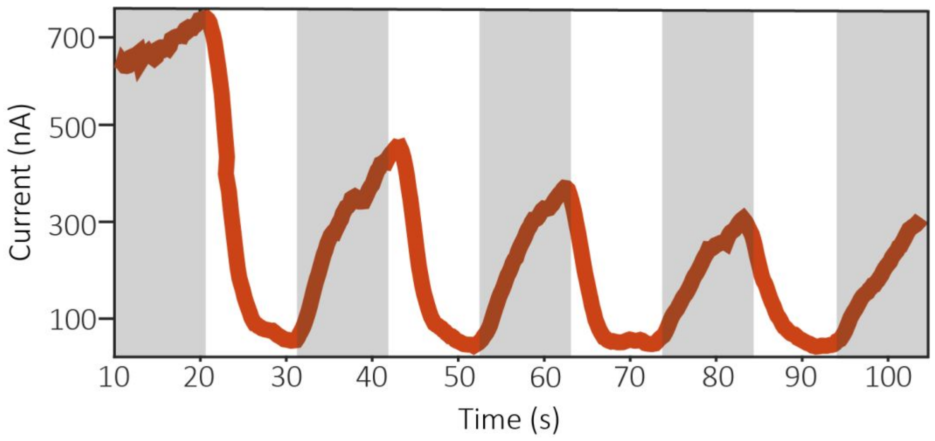

Figure S10: Time-dependent conductivity measurements showing negative photoconductivity of automatically fabricated nanowire device under white light illumination (white) and dark conditions (grey shading).

REFERENCES

(1) Gatrell, L. B.; Hoff, W. A.; Sklair, C. W. Robust Image Features: Concentric Contrasting Circles and Their Image Extraction. In *Cooperative Intelligent Robotics in Space II* SPIE **1992**, 1612, 235–244.

(2) Naimark, L.; Foxlin, E. Circular Data Matrix Fiducial System and Robust Image Processing for a Wearable Vision-Inertial Self-Tracker. In *Proceedings. International Symposium on Mixed and Augmented Reality* **2002**, 27–36.

(3) Moriyama, T.; Kochi, N.; Yamada, M.; Fukaya, N. Automatic Target-Identification with the Color-Coded-Targets. *Int. Arch. Photogramm. Remote Sensing, Beijing, XXI Congr. WG* **2008**, *1*, 39–44.

(4) Lee, K. B.; Kim, C. O. Marker Layout for Optimizing the Overlay Alignment in a Photolithography Process. *IEEE Trans. Semicond. Manuf.* **2019**, *32* (2), 212–219.

(5) Craig, A. B. *Understanding Augmented Reality Concepts and Applications*, 1st ed.; Morgan Kaufmann, Waltham, MA **2013**.

(6) Kato, H.; Billinghurst, M. Marker Tracking and HMD Calibration for a Video-Based Augmented Reality Conferencing System. In *Proceedings 2nd IEEE and ACM*

International Workshop on Augmented Reality (IWAR'99); IEEE Comput. Soc, **1999**, 85–94.

(7) Fiala, M. ARTag, a Fiducial Marker System Using Digital Techniques. In *2005 IEEE Computer Society Conference on Computer Vision and Pattern Recognition (CVPR'05),* 2, 590–596.

(8) Atcheson, B.; Heide, F.; Heidrich, W. CALTag: High Precision Fiducial Markers for Camera Calibration. *Vision, Model. Vis.* **2010**, 41–48.

(9) Wang, J.; Olson, E. AprilTag 2: Efficient and Robust Fiducial Detection. In *2016 IEEE/RSJ International Conference on Intelligent Robots and Systems (IROS),* **2016**, 4193-4198.

(10) Bergamasco, F.; Albarelli, A.; Rodola, E.; Torsello, A. RUNE-Tag: A High Accuracy Fiducial Marker with Strong Occlusion Resilience. In *CVPR 2011*, **2011**, 113–120.

(11) Olsson, L. Ö.; Andersson, C. B. M.; Håkansson, M. C.; Kanski, J.; Ilver, L.; Karlsson, U. O. Charge Accumulation at InAs Surfaces. *Phys. Rev. Lett.* **1996**, *76* (19), 3626–3629.

(12) Yang, Y.; Peng, X.; Kim, H.-S.; Kim, T.; Jeon, S.; Kang, H. K.; Choi, W.; Song, J.;

Doh, Y.-J.; Yu, D. Hot Carrier Trapping Induced Negative Photoconductance in InAs Nanowires toward Novel Nonvolatile Memory. *Nano Lett.* **2015**, *15* (9), 5875–5882.

(13) Han, Y.; Fu, M.; Tang, Z.; Zheng, X.; Ji, X.; Wang, X.; Lin, W.; Yang, T.; Chen, Q. Switching from Negative to Positive Photoconductivity toward Intrinsic Photoelectric Response in InAs Nanowire. *ACS Appl. Mater. Interfaces* **2017**, *9* (3), 2867–2874.
